# Novel Mutations in *COL6A3* That Associated With Peters’ Anomaly Caused Abnormal Intracellular Protein Retention and Decreased Cellular Resistance to Oxidative Stress

**DOI:** 10.3389/fcell.2020.531986

**Published:** 2020-11-10

**Authors:** Yue Li, Jing Zhang, Yiqin Dai, Yidan Fan, Jianjiang Xu

**Affiliations:** ^1^Eye Institute and Department of Ophthalmology, Eye and ENT Hospital, Fudan University, Shanghai, China; ^2^NHC Key Laboratory of Myopia, Fudan University, Shanghai, China; ^3^Shanghai Key Laboratory of Visual Impairment and Restoration, Shanghai, China

**Keywords:** Peters’ anomaly, anterior segment dysgenesis, *COL6A3*, endoplasmic reticulum stress, oxidative stress, apoptosis

## Abstract

Peters’ anomaly (PA) is a rare form of anterior segment dysgenesis characterized by central corneal opacity accompanied by iridocorneal or lenticulo-corneal adhesions. Although genetic mutations, particularly those affecting transcription factors that function in eye development, are known to cause PA, the etiology of this disease remains poorly understood. In this study, 23 patients with PA were recruited for panel sequencing. Four out of 23 patients were found to carry variants in known PA causal genes, PITX2 and PITX3. More importantly, two homozygous mutations (NM_057164: p.Val86Ala and p.Arg689Cys) in the *COL6A3* gene (collagen type VI alpha-3 chain) that correlated with the phenotype of type I PA were identified, and then validated by following whole-exome sequencing. The expression profile of the *COL6A3* gene in the cornea and the impact of the mutations on protein physiological processing and cellular function were further explored. It was shown that *COL6A3* presented relatively high expression in the cornea. The mutant COL6A3 protein was relatively retained intracellularly, and its expression reduced cellular resistance to oxidative stress through an enhanced endoplasmic reticulum stress response. Taken together, our findings expanded the known genetic spectrum of PA, and provided evidence for the involvement of *COL6A3* or collagen VI in ocular anterior segment development, thereby offering new insight for future investigations targeting PA.

## Introduction

The development of ocular anterior segment structures is a precisely coordinated process that is determined by both genetic and environmental factors. In humans, this process begins from week 6 of gestation, and is characterized by the formation of the lens placode from overlying surface ectoderm. The cornea is derived after lens detachment, while several waves of tissue invade the primary mesenchyme that lies behind the surface ectoderm, ultimately giving rise to an anterior epithelium, and a posterior endothelium, with the corneal stroma laying between these layers. A number of genes that include transcription factors, nuclear proteins, structural proteins, and enzymes are known to be involved in this sophisticated process, and defects in these key genes may lead to severe congenital anterior segment dysgenesis (ASD) (Sowden, 2007; Harissi-Dagher and Colby, 2008; Ma et al., 2019).

Peters’ anomaly (PA) is a rare form of ASD characterized by central corneal opacity, abnormal stromal structures, and defects in Descemet’s membrane, accompanied by iridocorneal or lenticulo-corneal adhesions (Peters, 1906; Stone et al., 1976; Bhandari et al., 2011; Nischal, 2015), and has an incidence of approximately 1.5 per 100,000 live births (Kurilec and Zaidman, 2014). Isolated PA can be categorized as type I characterized by the iridocorneal adhesions, or type II characterized by cataracts or lenticulo-corneal adhesions. Peters’ plus syndromes accompanied with short statue, cleft lip/palate, brachydactyly and facial dysmorphism are caused by *B3GLCT* mutations, which is not within the scope of this study. Most isolated PA cases are sporadic, while autosomal recessive or dominant patterns of inheritance have also been reported (Summers et al., 2008; Berker et al., 2009; Iseri et al., 2009). To date, most PA-related genes are those encoding transcription factors that are expressed in the eye or other structural components, such as *FOXE3, PAX6, PITX2, FOXC1*, and *CYP1B1* (Weisschuh et al., 2008; Arikawa et al., 2010; Mataftsi et al., 2011; Prokudin et al., 2014; Nischal, 2015), and these genes are also predominantly involved in several other types of ASD including aniridia, congenital cataract, and infantile glaucoma (Reis and Semina, 2011; Plaisancié et al., 2018; Ma et al., 2019). With the development of next-generation sequencing (NGS), the genetic spectrum of PA has been expanded to include *TFAP2A, HCCS, NDP, SLC4A11, FLNA*, and *COL4A1* (Deml et al., 2014; Weh et al., 2014). However, all these identified PA-causal genes can only explain a small proportion of the disease etiology, and most PA cases still lack a clear genetic diagnosis.

Structurally, the stroma makes up approximately 90% of the cornea, and is composed of collagen lamellae that are made by the epithelial cells and some stromal cells. The transparency of the cornea results from uniformity in both size and spacing of the collagen lamellae. Therefore, as an important structural component of the cornea, collagen defects have been suggested to cause several physiological abnormalities. For example, mutations in the *COL4A1* and *COL8A2* genes have been shown to induce PA, posterior polymorphous corneal dystrophy (PPCD), or corneal endothelial dystrophy (Biswas et al., 2001; Deml et al., 2014). It is possible that deficiencies in other collagen-encoding genes might also affect eye function, and exploring their roles in eye development might help to better elucidate the pathogenesis of PA and other types of ASD.

NGS-based panel sequencing of genomic regions of interest has been demonstrated to be a powerful approach for screening of disease-causal variants in clinical applications (Koboldt et al., 2013; Chaitankar et al., 2016; Gupta et al., 2017; Bick et al., 2019). Previously, a predesigned gene panel covering 801 candidate genes for various eye disorders was used successfully to identify the genetic spectra of corneal dystrophies in a Han Chinese population (Zhang et al., 2019c). In this study, 23 PA patients were recruited for genetic screening using the same panel, followed by whole-exome sequencing (WES) and functional investigations to confirm novel disease-causal genes. Our results expanded current knowledge of the range of genetic variants associated with PA; more importantly, we identified *COL6A3* as a potential candidate gene contributing to human type I PA.

## Materials and Methods

### Patients and Clinical Evaluation

This study was performed in accordance with the Declaration of Helsinki and was approved by the Ethics Committee of the Eye and ENT Hospital of Fudan University. We recruited 23 isolated PA patients with their unaffected parents, and 50 normal controls from the Eye and ENT Hospital, Fudan University, Shanghai, China. Written informed consent was obtained from all the participants. Diagnosis was based on specialized ophthalmologic examinations and corneal histological examination.

### Next-Generation Sequencing and Variant Analysis

Genomic DNA was extracted from peripheral blood, using the QIAGEN FlexiGene DNA Kit (Qiagen, Hamburg, Germany). Targeted region sequencing on a pre-designed panel including 801 genes was performed on all the 23 PA patients as previously described (Zhang et al., 2019b,c). One of them was further subjected to whole-exome sequencing (WES). Sequencing data that passed quality control were mapped to the hg19 assembly of the human genome using the BWA package. Variants were called using the GATK2 best practices pipeline^1^ and then annotated using the ANNOVAR package^2^.

Variant analysis was performed following an in-house bioinformatics pipeline (Zhang et al., 2019b,c). Briefly, common variants with minor allele frequency (MAF) ≥ 5% in East Asians in public databases (dbSNP144, 1000 Genome Project phase 3, Esp6500, and gnomAD) were removed. The prediction of the effect of amino acid substitution on protein function was performed through SIFT and Polyphen-2. Clustal Omega was used for analysis of sequence conservation.

### Animals

Balb/c mice (6–8 weeks old) were obtained from Shanghai Laboratory Animal Center, Chinese Academy of Sciences (CAS). The mice were euthanized by cervical dislocation, and the eyeballs were fixed and sectioned for further immunofluorescence staining. Total RNA was extracted from various murine tissues. All animal experimental procedures were approved by the Animal Care and Use Committee of Shanghai Medical College, Fudan University, and complied with the ARVO Statement for the Use of Animals in Ophthalmic and Vision Research.

### Immunofluorescence Staining

For tissues, murine eyeballs or human cornea were fixed in 4% paraformaldehyde (PFA), followed by dehydration in sucrose solutions. Cells were cultured on glass coverslips until 90–95% confluence, and then fixed in 4% PFA for 20 min. Next, samples were permeabilized with 0.03% Triton X-100, blocked with 3% BSA, and stained with primary antibodies (anti-COL6A3, 1:200, Sigma-Aldrich, United States; anti-FLAG, 1:2000, MBL, Japan; anti-BiP, 1:200, Abcam, United Kingdom). Lastly, the samples were stained with secondary antibody (Alexa Fluor 555-labeled donkey anti-rabbit IgG, 1:1000; and Alexa Fluor 488-labeled donkey anti-mouse IgG, 1:1000; both from Thermo Fisher Scientific, United States). The samples were examined by confocal microscopy (Leica Microsystems, Wetzlar, Germany). All the immunofluorescence image analyses were performed by ImageJ (NIH Image, United States, Version 1.52). For quantification of the BiP protein levels, we manually delineated at least ten non-overlapped areas of interest that included only FLAG-positive cells in every three immunofluorescence pictures from three independent experiments, to obtain the fluorescence intensity of FLAG and BiP, and then calculated the averaged fluorescence intensity ratio of BiP/FLAG of each picture. Then the averaged fluorescence intensity ratios of BiP/FLAG of each group were attained for comparison.

### Establishment of Cells Overexpressing Wide-Type or Mutant *COL6A3*

Immortalized human corneal epithelial cells (HCECs) were obtained and cultured as previously described (Zhang et al., 2019a). HEK293T cells (Shanghai Cell Bank, CAS) were cultured in DMEM (Gibco, Grand Island, NY, United States) supplemented with 10% FBS and 1% penicillin/streptomycin. A full-length wild-type human *COL6A3* cDNA clone (NM_057164), and a mutant *COL6A3* cDNA clone (NM_057164: c.257T > C and c.2065C > T) were obtained from YouBio Biometrics (Hunan, China), both containing a FLAG-tag on the C-terminus. The cDNA were separately cloned into the pHAGE-puro vector. HEK293T cells were co-transfected with the two pHAGE-puro plasmids, and the lentivirus packaging plasmids psPAX2 and pMD2.G for collecting lentiviruses carrying wide-type or mutant *COL6A3* cDNA. Then, HCECs were transfected with the two types of lentivirus respectively, followed by puromycin selection, to obtain cells stably expressing the transgenes (WT cells and MU cells). The un-transfected HCECs were regarded as negative control (NC).

### RNA Extraction and Quantitative Real-Time PCR

Total RNA was extracted using the RNA simple Total RNA Kit (TIANGEN, Beijing, China). The first strand cDNA was reverse transcribed using the FastQuant RT Kit (TIANGEN). Quantitative real-time PCR was subsequently conducted on the ABI ViiA 7 Real-Time PCR System (Thermo Lifetech, United States) using the QuantiNova SYBR Green PCR Kit (Qiagen). Primer sequences are shown in Supplementary Table 2.

### Western Blotting

Cellular proteins were extracted using cold RIPA lysis buffer (Beyotime, Shanghai, China) containing a protease inhibitor cocktail. Total proteins (30–50 μg) were separated on 10 or 12% SDS-polyacrylamide gels and then transferred onto PVDF membranes. The membranes were blocked with 5% non-fat dried milk, and then incubated with primary antibodies (anti-FLAG, 1:1000, Cell Signaling Technology; anti-BiP, 1: 1000, Abcam; anti-cleaved Caspase-3, 1:1000, Cell Signaling Technology; anti-beta-Actin-Peroxidase, 1:5000, Sigma-Aldrich) overnight at 4°C, followed by incubation with HRP-conjugated goat anti-rabbit IgG secondary antibody (1:1000, Cell Signaling Technology). Chemiluminescence solution (MilliporeSigma, United States) was used to reveal the bands. Representative blots from at least three independent experiments were shown.

### CCK-8 Assay

Human corneal epithelial cells were cultured in a 96-well plate (1 × 10^4^ cells per well), and treated with or without 200 μM of H_2_O_2_ (Sangon Biotech, Shanghai, China). At the indicated time points, 10 μL of the CCK-8 solution (Dojindo Laboratories, Kumamoto, Japan) mixed with 100 μL of the culture medium was added to each well. After a 2-h incubation, the absorbance was measured at 450 nm using a microplate reader (BioTek, United States).

### Analysis of Apoptosis by Flow Cytometry

Human corneal epithelial cells were seeded in 6-well plates (2 × 10^5^ cells/well). After 24 h, the cells were treated with 200 μM of H_2_O_2_ for 6 h. The cells were then collected and double stained with Annexin V-FITC and propidium iodide (PI) for 15 min in the dark (BD Pharmingen, San Diego, CA, United States), followed by flow cytometry that was performed on a Beckman CoulterMoFlo XDP. The percentages of early or late apoptotic cells were calculated using FlowJo software version 7.6 (BD Pharmingen). Representative images from at least three independent experiments were shown.

### Statistical Analysis

All quantitative data were expressed as means ± standard deviation of at least three independent experiments. Multiple group comparisons were conducted through one-way ANOVA followed by Bonferroni test with homoscedasticity, or Tamhane’s T2 test with heteroscedasticity. For two group comparisons, statistical significance was calculated by Student’s *t*-test. A *P*-value < 0.05 was considered significant. All the calculations were performed with SPSS 22.0 (IBM Corp. Armonk, NY, United States).

## Results

### Panel Sequencing Indicated a Mutation Detection Rate of 17.4% in Isolated PA Patients

**FIGURE 1:**
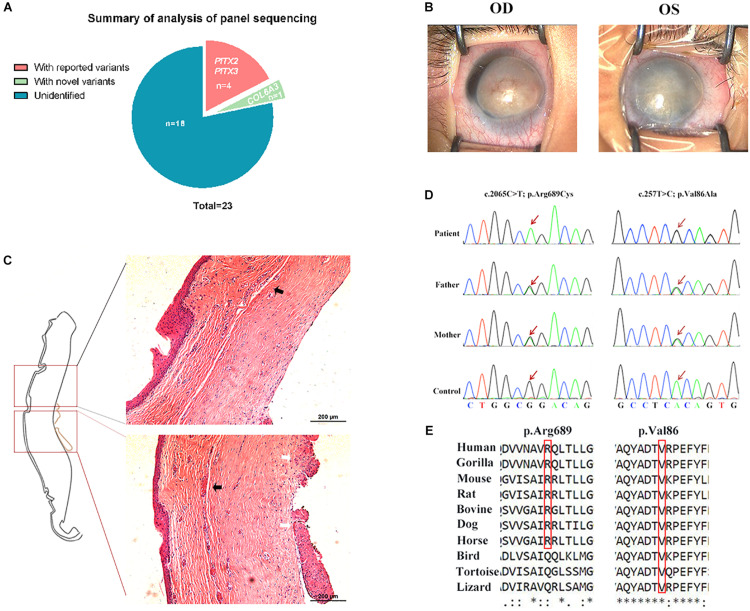
*COL6A3* was a novel candidate gene associated with type I Peters’ anomaly (PA). **(A)** Summary of the analysis of panel sequencing data for 23 patients with PA. Three reported variants in four patients, and one novel candidate gene were identified. **(B)** Photographs of corneas in the patient with type I PA. OD: oculus dexter (right eye), OS: oculus sinister (left eye). **(C)** Hematoxylin and eosin (HE)-stained corneal tissue sections; black arrows indicate the disordered lamellar arrangement with neoangiogenesis, and white arrows indicate the adhesion between cornea and iris. **(D)** Validation of the two novel homozygous mutations (NM_057164: c.2065C > T, p.Arg689Cys and c.257T > C, p.Val86Ala) by Sanger sequencing. **(E)** Conservation analysis on the two mutant residues across species.

Twenty-three patients with isolated PA were recruited, as well as their unaffected parents. The clinical characteristics are depicted in Table 1. Most patients (18/23) were diagnosed with type I PA, and 16 patients were affected bilaterally. Among them, nine patients have presented with glaucoma since the diagnoses or during the follow-ups. After panel sequencing, three reported PA-causal variants (*PITX2* NM_001204399: p.Pro64Arg, and *PITX3* NM_005029: p.Ala214fs, p.Gly219fs) (Weisschuh et al., 2006; Aldahmesh et al., 2011; Zazo et al., 2018) were found in four patients. The mutation detection rate was 17.4%, and all of them were affected bilaterally. More interestingly, we found one patient with type I PA carrying two novel homozygous mutations in the *COL6A3* gene (collagen type VI alpha-3 chain, NM_057164: p.Val86Ala and p.Arg689Cys), whose role in PA etiology remains unclear (Figure 1A and Table 2).

**TABLE 1:** Summary of clinical characteristics of recruited patients.

	***n* = 23**
**Average age at first surgery (month)**	12.3 ± 9.6
**Male**	13(57%)
**Bilateral cornea affected**	16(70%)
**Diagnosis**	
Type I Peters’ anomaly	18 (78%)
Type II Peters’ anomaly	5 (22%)
**With glaucoma**	9 (39%)
**Discoveries in DNA sequencing**	
Known mutations	*PITX2* (1) *PITX3* (3)
Novel candidate gene	*COL6A3* (1)

**TABLE 2:** Summary of analysis of panel sequencing.

**Gene**	**Nucleotide change**	**Zygosity**	**Protein change**	**Mutation type**	**PolyPhen2**	**SIFT**	**1000G**	**gnom AD**
**With reported variants**								
*PITX2* NM_001204399	c.191C > G	Heterozygous	p.Pro64Arg	Missense	Damaging	Damaging	0	0
*PITX3* NM_005029	c.640-656del	Heterozygous	p.Ala214fs	Frameshift	N/A	N/A	0	0
*PITX3* NM_005029	c.656-657insGCCC	Heterozygous	p.Gly219fs	Frameshift	N/A	N/A	0	0
	TGCAGGGCCTGGG							
**Novel candidate gene**								
*COL6A3* NM_057164	c.2065C > T	Homozygous	p.Arg689Cys	Missense	Damaging	Damaging	0.004	0.0019
*COL6A3* NM_057164	c.257T > C	Homozygous	p.Val86Ala	Missense	Damaging	Tolerant	0.0089	0.0152

### Whole-Exome Sequencing Confirmed the Association of *COL6A3* Mutations With Type I Peters’ Anomaly

The patient carrying *COL6A3* mutations was a Han Chinese boy with bilateral corneal opacity (Figure 1B), born to healthy parents after an uneventful pregnancy. The ultrasound bio-microscopy examination revealed bilateral corneal degeneration, corneal thickening, and a shallow anterior chamber, accompanied with iridocorneal adhesion and almost closed anterior angles. After penetrating keratoplasty of his left eye, corneal histological examination also supported the diagnosis of type I PA (Figure 1C). During the follow-ups, the patient did not develop glaucoma. WES was performed on this patient for further validation. In accordance with previous panel sequencing results, considering parents’ genotype and gene function still prioritized that *COL6A3* was likely to be associated with this PA patient (Supplementary Table 1).

The *COL6A3* gene encodes the alpha-3 chain of collagen VI, which is important for organizing matrix components. These two *COL6A3* mutations were both located within the von Willebrand factor type A domains that are involved in multiprotein complexes (Figure 1D). Sequence conservation analysis showed that the two residues were quite conserved among vertebrates, especially among mammals (Figure 1E). Taken together, these findings suggested that homozygous mutations in *COL6A3* could be novel candidate variants associated with human PA.

### *COL6A3* Was Highly Expressed in the Cornea

**FIGURE 2:**
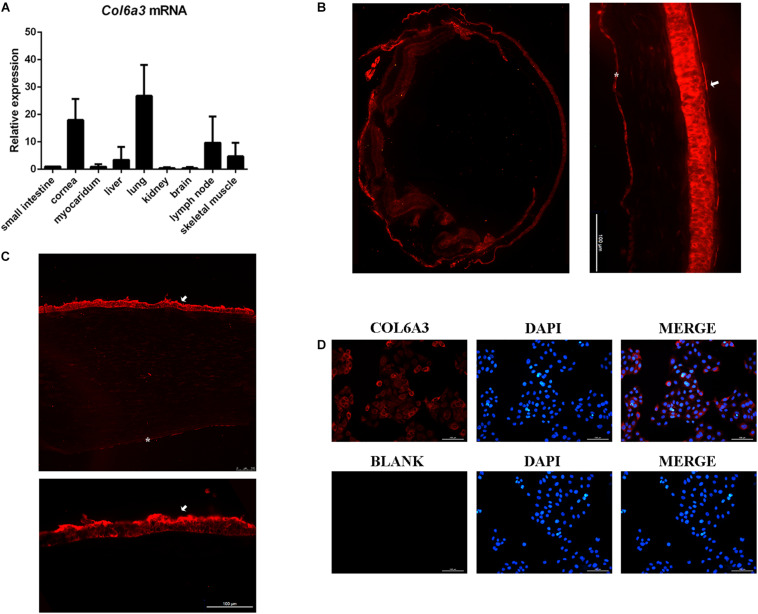
Relatively high expression of *COL6A3* in the cornea. **(A)** Expression profiles of *Col6a3* mRNA in various murine tissues, as determined by RT-qPCR. Immunofluorescence images of COL6A3 protein distribution (red) in the mouse eye **(B)** and human cornea **(C)**. White arrows indicate the corneal epithelium, while the white asterisks indicate the corneal endothelium. **(D)** Immunofluorescence staining of the COL6A3 protein (red) in a human corneal epithelial cell line; nuclear DNA was counterstained with DAPI (blue).

To obtain insight into the expression features of the *COL6A3* gene, we compared its expression profile in various mouse tissues using RT-qPCR, and found that it was highly expressed in the lung, cornea, and lymph nodes (Figure 2A). Immunofluorescence staining showed that the COL6A3 protein was predominantly localized to the corneal epithelium, with limited expression in the corneal stroma and endothelium. Subtle protein expression of Col6a3 was also observed in the mouse iris, ciliary body, and retina (Figures 2B,C). Immunofluorescence staining in the immortalized human corneal epithelial cells (HCECs) confirmed abundant COL6A3 expression, particularly in the cytoplasm (Figure 2D).

**FIGURE 3:**
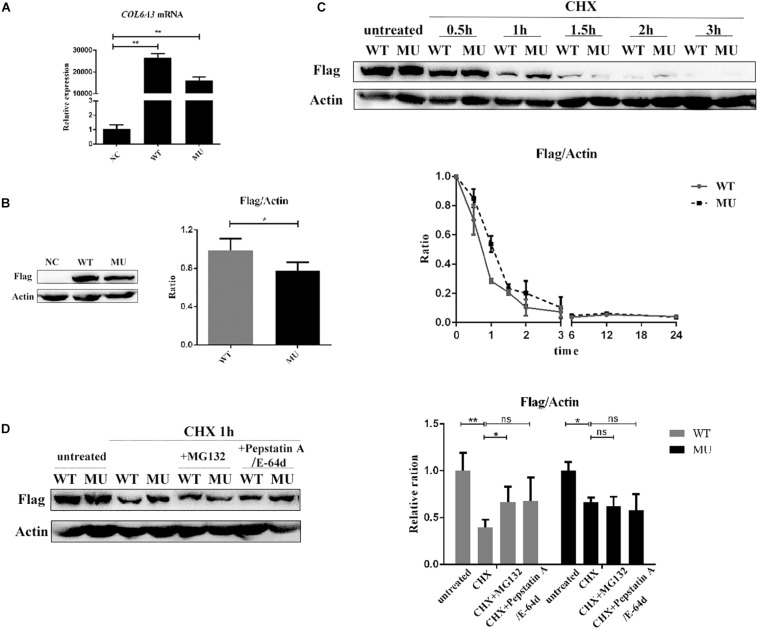
The mutant COL6A3 protein was retained intracellularly. **(A)** Validation of the overexpression of wide-type or mutant *COL6A3* in human corneal epithelial cell lines (HCECs) after transduction with lentiviral particles by RT-qPCR (WT vs un-transfected: *P* = 0.002; MU vs un-transfected: *P* = 0.004). The *P*-values were calculated by Student’s t-test for two group comparisons. **(B)** Validation of the overexpression of wide-type or mutant *COL6A3* in human corneal epithelial cell lines (HCECs) after transduction with lentiviral particles by western blotting (WT vs MU: *P* = 0.020). The COL6A3 protein level of WT or MU cells was visualized using an anti-FLAG antibody. The *P*-values were calculated by Student’s *t*-test for two group comparisons. **(C)** WT and MU cells were treated with 5 μg/mL cycloheximide (CHX) for 0.5, 1, 1.5, 2, and 3 h, respectively. Quantification of intracellular COL6A3 protein levels in WT or MU cells were determined by western blotting using an anti-FLAG antibody, and relative COL6A3 protein quantities were plotted and illustrated over time. **(D)** WT and MU cells were treated with 10 μM Pepstatin A/E-64d or 10 μM MG132, in the presence of 5 μg/mL CHX for 1 h. The intracellular COL6A3 protein levels in WT or MU cells were determined by western blotting using an anti-FLAG antibody. The graphs showed quantification of intracellular COL6A3 protein from western blotting bands; values were the average with standard deviation obtained from three independent experiments (WT group: untreated vs CHX: *P* = 0.004; CHX vs CHX + MG132: *P* = 0.043; MU group: untreated vs CHX: *P* = 0.040). The *P*-values were calculated by one-way ANOVA test, followed by Bonferroni test. NC: the un-transfected HCECs. *P < 0.05, **P < 0.01, ***P < 0.001.

Next, the major *COL6A3* transcript expressed in the human cornea, NM_057164 was used as a template to generate *COL6A3* overexpression constructs (Supplementary Figure 1). Then HCECs were transduced with lentiviral vectors expressing either the wide-type or mutant *COL6A3* cDNA. Both constructs induced the high expression of the transgenes, as determined by RT-qPCR and western blotting (WT vs un-transfected: *P* = 0.002; MU vs un-transfected: *P* = 0.004, Figure 3A; WT vs MU: *P* = 0.020, Figure 3B).

### Mutant COL6A3 Protein Was Retained Intracellularly

As a key constitution of collagen VI, the COL6A3 protein is known to actively participate in collagen assembly, as well as collagen secretion to the extracellular matrix (ECM). Therefore, we first investigated whether the identified mutations affected the normal physiological processing of the COL6A3 protein. HCECs transfected with either wide-type (WT) or mutant (MU) cDNA were treated with cycloheximide (CHX, 5 μg/mL) to suppress protein synthesis, for comparing the intracellular elimination rates of the COL6A3 protein. The results showed a markedly slower elimination rate for mutant COL6A3 than for its wide-type form, particularly within the first hour of CHX treatment (Figure 3C). Both mutant and wide-type COL6A3 were completely eliminated after 3 h of CHX treatment.

We next examined whether these overexpressed COL6A3 proteins could be normally processed by intrinsic cellular degradation machineries, such as autophagy or the ubiquitylation pathway. In the WT group, treatment with specific inhibitors for autophagy (Pepstatin A, 10 μM; and E-64, 10 μM, both from Sigma-Aldrich) or ubiquitylation (MG132, 10 μM, Selleck, United States) efficiently restored the reduction of intracellular COL6A3 protein levels due to CHX treatment (untreated vs CHX: *P* = 0.004; CHX vs CHX + inhibitors: *P* = 0.043 and *P* = 0.136 respectively, Figure 3D). However, it was intriguing to find that the mutant COL6A3 protein was not sensitive to ubiquitylation- or autophagy-associated degradation. Compared with cells treated with CHX alone, the amount of intracellular COL6A3 protein in the MU group did not show a significant increase in the presence of MG132 (*P* = 0.649). When autophagy inhibitors were applied, the level of the mutant COL6A3 protein even slightly decreased (*P* = 0.379, Figure 3D). Therefore, these results suggested that the mutant COL6A3 might be retained intracellularly, in line with its observed lower elimination rate.

### Intracellular Retention of Mutant COL6A3 Increased Endoplasmic Reticulum Stress

**FIGURE 4:**
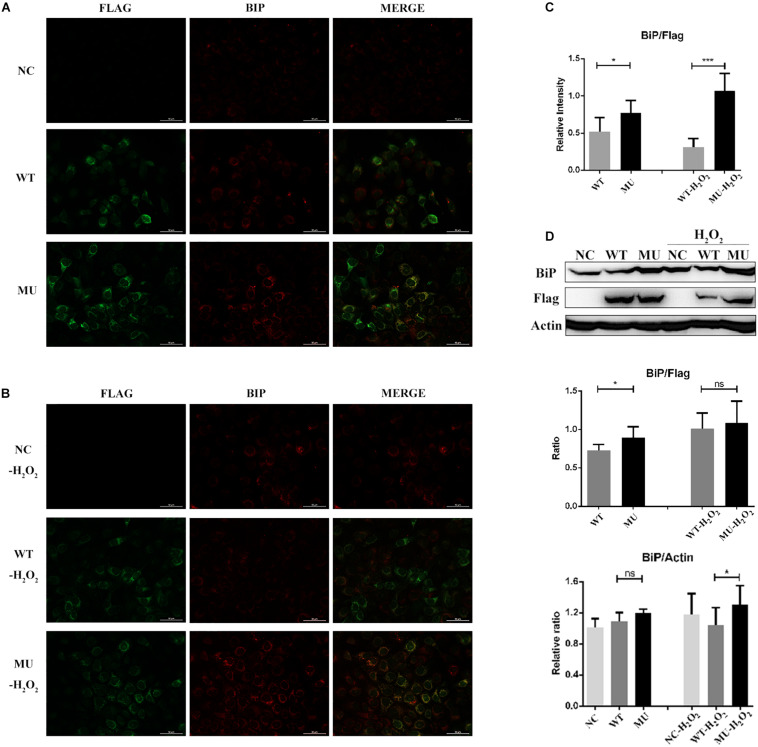
Endoplasmic reticulum stress was enhanced in cells overexpressing mutant *COL6A3*. **(A)** Immunofluorescence staining of BiP (red) as well as COL6A3 (green) proteins in un-transfected, WT, and MU cells under normal conditions. **(B)** Immunofluorescence staining of BiP (red) as well as COL6A3 (green) in un-transfected, WT, and MU cells upon 200 μM of H_2_O_2_ exposure for 1.5 h. **(C)** Column graph comparing the ratio of BiP/COL6A3 levels in FLAG-positive cells between WT and MU groups exposed or not exposed to 200 μM of H_2_O_2_. Values were derived from immunofluorescence staining images. WT vs MU: *P* = 0.037; WT-H_2_O_2_ vs MU-H_2_O_2_: *P* = 0.000003. The *P*-values were calculated by Student’s t-test for two group comparisons. **(D)** Western blotting for the detection of the BiP protein among un-transfected, WT, and MU HCECs exposed or not exposed to 200 μM of H_2_O_2_. The quantitative graph displayed the ratio of BiP/beta-Actin levels of the three groups (based on the ratio of un-transfected group without H_2_O_2_ exposure), and BiP/FLAG levels between WT and MU cells (BiP/FLAG, WT vs MU: *P* = 0.0198; BiP/Actin, WT-H_2_O_2_ vs MU-H_2_O_2_: *P* = 0.0102). The *P*-values were calculated by Student’s *t*-test for two group comparisons. NC: the un-transfected HCECs. *P < 0.05, ***P < 0.001.

Subcellular localization information obtained from the COMPARTMENTS database indicated that the intracellular COL6A3 protein was predominantly localized to the endoplasmic reticulum (ER). Given the emerging evidence of ER stress in relation with several corneal diseases (Gould et al., 2007; Chen et al., 2013; Han et al., 2016; Okumura et al., 2017; Wang et al., 2017), we next examined whether cells containing the retained mutant COL6A3 protein presented features of ER stress. When compared with the WT group, the level of BiP, an ER stress marker, in FLAG-positive cell (over-expressing COL6A3 protein), was significantly higher in the MU group (*P* = 0.037; Figures 4A,C). Moreover, the difference was even greater when the cells were exposed to 200 μM of H_2_O_2_ (*P* = 0.000003; Figures 4B,C). The higher BiP expression in the MU group was also supported by western blotting analysis, although the difference of the BiP/FLAG ratio did not reach statistical significance, probably due to the inclusion of FLAG-negative cells (BiP/FLAG, WT vs MU: *P* = 0.0198; BiP/Acitn, WT-H_2_O_2_ vs MU-H_2_O_2_: *P* = 0.0102; Figure 4D).

### Mutant COL6A3 Affected Cell Survival and Apoptosis

**FIGURE 5:**
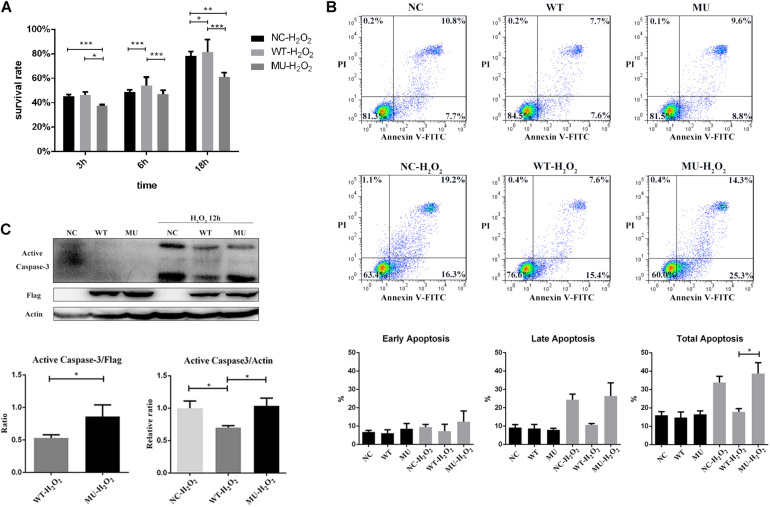
Cell survival was decreased and apoptosis was increased in cells overexpressing mutant *COL6A3*. **(A)** Cell viability of un-transfected, WT, and MU cells in response to H_2_O_2_ stimulation (200 μM) for 3, 6, and 18 h, respectively, as determined by CCK-8 assay. For cell viability comparisons after 6 h treatment with homoscedasticity, the *P*-values were calculated by one-way ANOVA test, followed by Bonferroni test. For cell viability comparisons after 3 h and 18 h treatment with heteroscedasticity, the *P*-values were calculated through Tamhane’s T2 test. **(B)** Representative scattergram of apoptotic HCECs. The un-transfected, WT, and MU groups stimulated or not stimulated with H_2_O_2_ for 6 h were assessed for apoptosis through flow cytometry based on Annexin V/propidium iodide (PI) double staining. The percentage of early/late apoptotic cells (%) below in each individual group was calculated from three independent experiments (WT vs MU in total apoptosis with H_2_O_2_ stimulation: *P* = 0.032). The *P*-values were calculated by one-way ANOVA test, followed by Bonferroni test. **(C)** Detection of apoptosis by caspase-3 activation. Cells treated with 200 μM of H_2_O_2_ for 12 h were harvested and cell lysates were prepared for western blotting to detect the levels of active caspase-3. Beta-actin and FLAG was used as the loading control. The column graph showed quantification of active caspase-3 protein from western blotting bands; values were the average with standard deviation obtained from three independent experiments (Active Caspase-3/FLAG: WT-H_2_O_2_ vs MU-H_2_O_2_: *P* = 0.042; Active Caspase-3/Actin: WT-H_2_O_2_ vs un-transfected-H_2_O_2_: *P* = 0.030; WT-H_2_O_2_ vs MU-H_2_O_2_: *P* = 0.026). The *P*-values were calculated through Student’s t-test and Tamhane’s T2 test. NC: the un-transfected HCECs. *P < 0.05, **P < 0.01, ***P < 0.001.

Because intracellular retention of mutant COL6A3 triggered ER stress, next we explored whether cell survival was influenced. Under normal culture conditions, no evident difference was observed in cell morphology or growth rate either among un-transfected HCECs, or those WT or MU cells. We further examined cell viability in response to H_2_O_2_ treatment for different periods (Figure 5A). Compared with the un-transfected group, the WT cells were more viable and resistant to H_2_O_2_ stimulation after 6 h, or 18 h of treatment (*P* = 0.0002 and *P* = 0.021, respectively). In contrast, the overexpression of mutant COL6A3 led to a remarkable decrease in the cell survival rate earlier after 3 h (*P* = 0.025), while the difference in cell viability between MU and WT groups was more evident in response to H_2_O_2_ exposure for 6 h and 18 h (*P* = 0.00014 and P = 0.000005, respectively).

Analysis of apoptosis by Annexin V/PI double staining also indicated that the overexpression of wide-type COL6A3 protected cells against H_2_O_2_-induced apoptosis. Compared with the un-transfected group, the proportion of apoptotic cells in the WT group decreased by nearly 20%; the difference was more pronounced for late apoptotic cells, but did not reach significance (Figure 5B). The apoptotic rate in the MU cells was slightly higher than that of the un-transfected group, implying that mutations may neutralize the protective effect of overexpressed wide-type COL6A3 against H_2_O_2_-induced apoptosis. Compared with WT cells, the percentage of total apoptosis in MU cells was significantly higher with H_2_O_2_ stimulation (*P* = 0.032). Consistently, the level of active caspase-3 was significantly lower in the WT group than in either the MU group or the un-transfected group (Active Caspase-3/FLAG: WT-H_2_O_2_ vs MU-H_2_O_2_: *P* = 0.042; Active Caspase-3/Actin: WT-H_2_O_2_ vs un-transfected-H_2_O_2_: *P* = 0.030; WT-H_2_O_2_ vs MU-H_2_O_2_: *P* = 0.026; Figure 5C).

Taken together, this study suggested *COL6A3* as a novel candidate gene contributing to human PA. The newly identified *COL6A3* mutations induced intracellular protein retention, and enhanced the ER stress response, thereby leading to reduced cell viability under oxidative stress.

## Discussion

In this study, panel screening of 23 PA patients found three reported variants in established PA causal genes. The mutation detection rate was only 17.4% in isolated PA patients, which was much lower than the detection rate of other ocular diseases with strong genetic components involved (Patel et al., 2019; Zhang et al., 2019c). Our findings revealed that current knowledge on the genetic contributors associated with isolated PA was far from understood, and thereby genetic screening on PA patients might be urgent needed.

Importantly, followed by WES validation, we demonstrated *COL6A3* as a novel candidate gene contribution to human type I PA. The mutant COL6A3 protein exhibited a markedly slower intracellular elimination rate than the wide-type form, and was not sensitive to the typical intracellular degradation machineries, indicating that mutations in the COL6A3 protein might lead to its abnormal intracellular retention. Subsequently, an enhanced ER stress response, followed by decreased cellular resistance to oxidative stress was observed in cells expressing the mutant form of COL6A3.

Collagen VI beaded microfibrils are found in the ECM of virtually all tissues. The domain structure of collagen VI is encoded by three genes, *COL6A1, COL6A2*, and *COL6A3*. Three additional new chains that only existed in mammals, encoded by *COL6A4*, *COL6A5*, and *COL6A6*, share a common evolutionary history with *COL6A3* (Fitzgerald et al., 2008). Among them, the longest alpha-3 chain encoded by *COL6A3* is more essential for the stability and microfibril assembly of collagen VI molecules (Lamand et al., 1998; Lamandé et al., 2006). Previously, dominant and recessive mutations in these key genes have been found in a spectrum of muscle disorders ranging from the mild Bethlem myopathy to the severe Ullrich congenital muscular dystrophy. In addition to muscle defects, affected patients or mice also present with skin and tendon features, underlining the important roles of collagen VI in the ECM of numerous other tissues (Bönnemann, 2011; Bernardi and Bonaldo, 2013; Pan et al., 2013). It is quite interesting to find that almost all the reported mutations that associated with muscular dystrophy were located after the 10th exon in the longest transcript of *COL6A3*. However, the dominant transcript isoforms in the cornea do not contain these exons (shown in Supplementary Figure 1). The mutation associated with collagen VI-related myopathy usually caused the shortened alpha-3 chain, followed by the abnormal secretion, and even led to the complete absence of collagen VI. At the same time, the mutations also led to the mitochondrial dysfunction, defective autophagy, and increased apoptosis, all participating in the pathogenesis of collagen VI muscular dystrophy (Bönnemann, 2011; Bernardi and Bonaldo, 2013). Therefore, different mutations in *COL6A3* may cause abnormalities in different organs/tissues, differing in the spatial expression abundance of different transcripts.

Collagen VI is shown to be abundantly expressed in the cornea (Engvall et al., 1986; Zimmermann et al., 1986; Delaigue et al., 1995) and *in vitro* studies have suggested that it is actively involved in corneal development. For example, Takahashi et al. (1993) showed that corneal proteoglycans, which are responsible for maintaining corneal hydration, rigidity, and transparency are associated with collagen VI in the developing rabbit cornea. Doane et al. (1992, 1996, 1998) demonstrated that collagen VI localization not only exhibits spatial and temporal variations during corneal development, but also promotes the adhesion and spreading of corneal fibroblasts, implying an important role for collagen VI in cell-matrix and matrix-matrix interactions during corneal stroma development. A recent large-scale genome-wide association study demonstrated the contribution of *COL6A1* small nucleotide polymorphisms to keratoconus susceptibility, linking variation in the collagen VI encoding gene with a specific ocular disease for the first time (Khawaja et al., 2019). Here, the identification of novel *COL6A3* mutations associated with human PA in this study agrees well with previous reports that have highlighted the critical role of collagen VI in corneal development.

The ER is a membranous tubular network with functions in cellular protein biosynthesis and lipid and calcium homeostasis. Genetic defects, as well as a wide range of external stimuli, can induce the accumulation of misfolded or unfolded proteins in the ER, thereby triggering the unfolded protein response and inducing apoptosis. Indeed, intracellular accumulation of mutant proteins has been observed in many corneal dystrophies (Gould et al., 2007; Jun et al., 2011; Bernardi and Bonaldo, 2013; Chen et al., 2013; Allen et al., 2016; Han et al., 2016; Okumura et al., 2017; Wang et al., 2017), highlighting that a “mutant protein accumulation-increased ER stress-reduced cell viability” pathway might be a critical contributor to disease pathogenesis. In addition, the alpha-5 and alpha-6 chains of collagen VI were shown to be retained inside the cells under a particular circumstance. The alpha-3 chain may behave like the alpha-5/6 chain, as they share much similarity in structure and function (Fitzgerald et al., 2008). In the current study, given the known subcellular localization of COL6A3 in the ER, we hypothesized that there might be an interplay between the novel PA-related *COL6A3* mutations and ER function. In agreement with our assumption, we demonstrated that the mutations led to intracellular protein retention and rendered the cells more vulnerable to ER stress, thereby reducing cellular resistance to oxidative stress.

It is worth noting that if only considering variant allele frequency, it is questionable to conclude that the two COL6A3 variants are causal, since their allele frequencies in the general population were not low enough (1.5% and 0.1% MAF in East Asians), making it difficult to classify them as pathogenic causal variants for PA. However, PA is a subtype of ASD, and there are several pieces of evidence already showing that ASD phenotypes often exhibit variable expressivity and incomplete penetrance pointing to a complex etiology, implying that reduced penetrance might be a possibility. Therefore, in order to further support the contribution of the mutant protein to PA pathogenesis, we presented intensive functional data to support the variants being detrimental. Our results clearly demonstrated the variants’ pathogenic impacts on cellular function, leading to intracellular protein retention, and rendering cells more vulnerable to oxidative stress. Thus, we suggested that the observation of several carriers in the general population might be due to the decreased penetrance of this disease, which was also observed in other PA-related genes (Bejjani et al., 2000).

Our study also had several limitations. First, the data presented here can only explain the effect of mutant COL6A3 on exacerbating ER stress, and the ensuing decreased cell viability. However, the protective effect of the abundant level of wide-type COL6A3 protein shown in this study, as well as the associated underlying mechanisms, remains unclear. In fact, there were some previous studies showing the cytoprotective role of collagen VI via interacting apoptosis and oxidative damage in muscles (Irwin et al., 2003; Bönnemann, 2011; Bernardi and Bonaldo, 2013). Besides, some other studies have found that a lack of collagen VI in neural cells led to spontaneous apoptosis and defective autophagy, indicating its protective role during physiological aging or under stress (Cheng et al., 2009, 2011; Cescon et al., 2016). They are all consistent with the results in this study to some extent, and the protective effect of COL6A3 and its mechanisms need to be further investigated. Second, we only examined the influence of mutant COL6A3 within the cell, and it remains unclear whether the mutations also affect collagen secretion into the ECM. Finally, since PA is a quite rare disease, we were unable to find recurrent *COL6A3* mutations in other unrelated cases at this moment. It is possible that only one variant was pathogenic, or their combined effect contributed to disease pathogenesis. Given the real mutated situation in this PA patient, and to mimic it, we investigated the two COL6A3 mutations together at this stage. Also, we cannot fully exclude the possibility that the two mutations identified here were just the tags of another real causal variant, and thereby further screening on this interested region in more PA patients is needed.

In conclusion, we obtained a mutation detection rate of 17.4% in isolated PA patients, highlighting the urgent need of expanding the genetic spectrum of PA through screening more patients. Also, we suggested *COL6A3* as a novel candidate gene contributing to human PA. Mutations in the COL6A3 protein resulted in its intracellular retention, and reduced cell viability through an enhanced ER stress response, indicating that these effects might be involved in PA pathogenesis. To the best of our knowledge, this is the first study showing the critical role of *COL6A3* in ocular segment dysgenesis, i.e., PA, and our findings may aid in the future development of gene therapy for this rare disease.

## Data Availability Statement

The datasets generated for this study can be found in the figshare database at 10.6084/m9.figshare.1010144.

## Ethics Statement

The studies involving human participants were reviewed and approved by the Ethics Committee of Eye and ENT Hospital of Fudan University. Written informed consent to participate in this study was provided by the participants’ legal guardian/next of kin. The animal study was reviewed and approved by Animal Care and Use Committee of Shanghai Medical College, Fudan University. Written informed consent was obtained from the minor(s)’ legal guardian/next of kin for the publication of any potentially identifiable images or data included in this article.

## Author Contributions

JX and JZ designed the study. YL and JZ performed the experiments, analyzed the data, and drafted the manuscript. YL, YF, and YD collected the clinical data. JX, YD, and YF revised the manuscript. All authors read and approved the final manuscript.

## Conflict of Interest

The authors declare that the research was conducted in the absence of any commercial or financial relationships that could be construed as a potential conflict of interest.

## Footnotes

1 https://software.broadinstitute.org/gatk/

2 www.openbioinformatics.org/annovar/

## Supplementary Material

Supplementary Figure 1 Screening of the major *COL6A3* isoform that expressed in human cornea. **(A)** The electrophoresis results of PCR products from different *COL6A3* isoforms in six independent human cornea samples. The product length of NM_004369 was 180 bp, while the one of NM_057164/NM_057165 was 197 bp. **(B)** Relatively higher expression of *COL6A3* isoform NM_057164/NM_057165 in human cornea detected by RT-qPCR (*N* = 4, *P* = 0.017). **(C)** The structural difference between the two *COL6A3* isoform-coding sequences, and locations of the *COL6A3* mutations related with myopathy and PA in this study. The *P*-values were calculated by Student’s *t*-test. **P* < 0.05.

Supplementary Table 1 Variants identified by whole-exome sequencing.

Supplementary Table 2 Primers used in PCR.
