# Supplementary figures and images for: Novel Mutations in COL6A3 That Associated With Peters’ Anomaly Caused Abnormal Intracellular Protein Retention and Decreased Cellular Resistance to Oxidative Stress
Source: Front Cell Dev Biol. 2020 Nov 10;8:531986. doi: 10.3389/fcell.2020.531986 (PMC7693641; doi:10.3389/fcell.2020.531986)

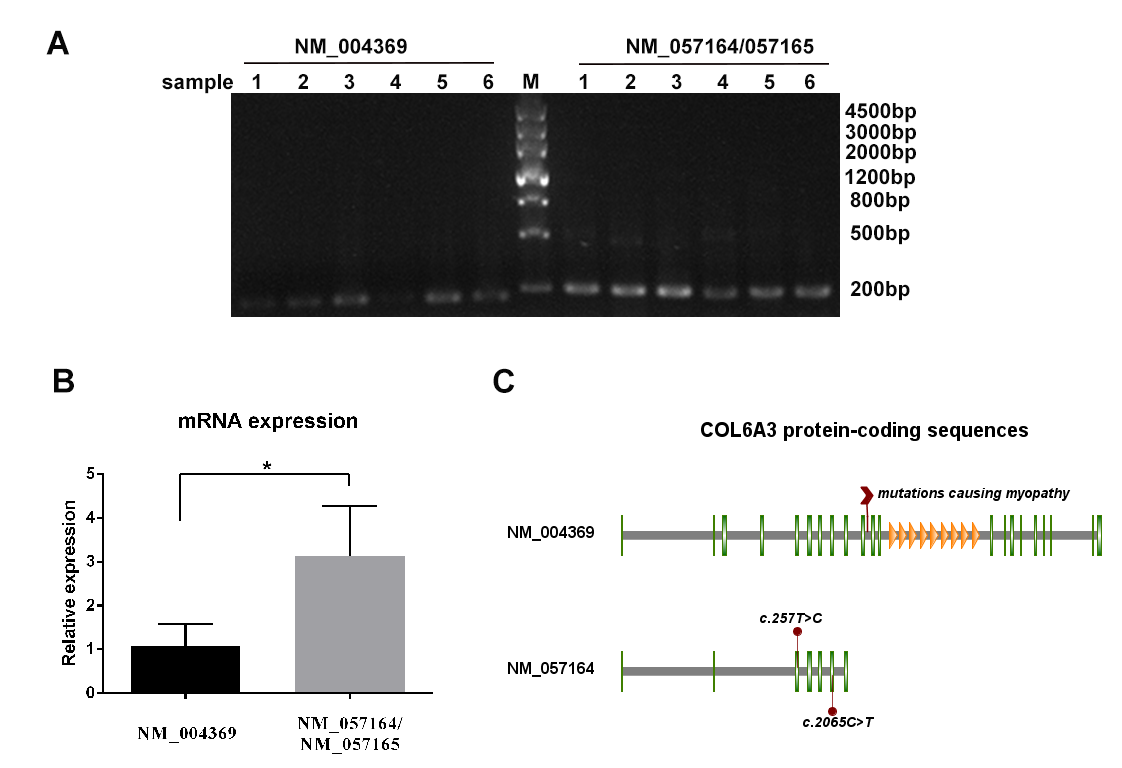

Supplement: Supplementary Figure 1 — Screening of the major COL6A3 isoform that expressed in human cornea. (A) The electrophoresis results of PCR products from different COL6A3 isoforms in six independent human cornea samples. The product length of NM_004369 was 180 bp, while the one of NM_057164/NM_057165 was 197 bp. (B) Relatively higher expression of COL6A3 isoform NM_057164/NM_057165 in human cornea detected by RT-qPCR (N = 4, P = 0.017). (C) The structural difference between the two COL6A3 isoform-coding sequences, and locations of the COL6A3 mutations related with myopathy and PA in this study. The P-values were calculated by Student’s t-test. *P < 0.05. [file Image_1.TIF]
