# Supplementary material for: Novel Mutations in COL6A3 That Associated With Peters’ Anomaly Caused Abnormal Intracellular Protein Retention and Decreased Cellular Resistance to Oxidative Stress
Source: Front Cell Dev Biol. 2020 Nov 10;8:531986. doi: 10.3389/fcell.2020.531986 (PMC7693641; doi:10.3389/fcell.2020.531986)
Supplement: Supplementary Table 1 — Variants identified by whole-exome sequencing. [file Table_1.DOCX]

**Supplementary Table S1 Variants identified by whole-exome sequencing**

| **Gene** | **Nucleotide change** | **Zygosity** | **Protein**  **change** | **Mutation type** | **PolyPhen2** | **SIFT** | **1000G** | **gnom**  **AD** |
| --- | --- | --- | --- | --- | --- | --- | --- | --- |
| *COL6A3* NM_057164 | c.2065C>T | Homozygous | p.Arg689Cys | Missense | Damaging | Damaging | 0.004 | 0.0019 |
| *COL6A3* NM_057164 | c.257T>C | Homozygous | p.Val86Ala | Missense | Damaging | Tolerant | 0.0089 | 0.0152 |
| *IQCA1* NM_024726 | c.1382G>A | Homozygous | p.Arg461His | Missense | Benign | Tolerant | 0.0188 | 0.0146 |
| *SRPRB* NM_021203 | c.754A>G | Homozygous | p.Arg252Gly | Missense | Benign | Tolerant | 0.0367 | 0.0351 |
| *DAB2* NM_001343 | c.1757C>T | Homozygous | p.Thr586Ile | Missense | Benign | Damaging | 0.0427 | 0.0341 |
