# Supplementary material for: Novel Mutations in COL6A3 That Associated With Peters’ Anomaly Caused Abnormal Intracellular Protein Retention and Decreased Cellular Resistance to Oxidative Stress
Source: Front Cell Dev Biol. 2020 Nov 10;8:531986. doi: 10.3389/fcell.2020.531986 (PMC7693641; doi:10.3389/fcell.2020.531986)
Supplement: Supplementary Table 2 — Primers used in PCR. [file Table_2.DOCX]

**Supplementary Table S2 Primers used in PCR**

| **Primer names** | **Sequence (5’>3’)** |
| --- | --- |
| *COL6A3* NM_057164 p. Arg689Cys Forward | CTCTCAGAGATGACCTGTTGGAC |
| *COL6A3* NM_057164 p. Arg689Cys Reverser | CGTTGATATGAAATTGCTGCTCTGA |
| *COL6A3* NM_057164 p. Val86Ala Forward | CTGAACTCGTGAATAGGTTGTTACG |
| *COL6A3* NM_057164 p. Val86Ala Reverse | ATGAGGGTGTCCTTTAAATGGAGTT |
| Mouse *Gapdh* Forward | CATCACTGCCACCCAGAAGACTG |
| Mouse *Gapdh* Reverse | ATGCCAGTGAGCTTCCCGTTCAG |
| Mouse *Col6a3* Forward | CCACGGAAGTTCACGTAAACAAA |
| Mouse *Col6a3* Reverse | AAACTGCACCAAGCCAACAC |
| *COL6A3* NM_004369 Forward | ATTCATCCGTGAGTCCAGAAAGG |
| *COLA63* NM_004369 Reverse | GCTGAGTTCCAATTGGGAGTTTC |
| *COL6A3* NM_057164/ NM_057165 Forward | TGTTCTCGGTGAGCACCTTCC |
| *COL6A3* NM_057164/ NM_057165 Reverse | CATGGCCTTTGAGCCTGTTATTT |
| *COL6A3* Forward | TTTCATCTTCCAAGCCAAGAACG |
| *COL6A3* Reverse | CAGAAACACCACGTCCTTTTCAC |
| Human *GAPDH* Forward | GTCTCCTCTGACTTCAACAGCG |
| Human *GAPDH* Reverse | ACCACCCTGTTGCTGTAGCCAA |
